# Restructuring ENT out-patient services during the coronavirus disease 2019 pandemic – an iterative approach

**DOI:** 10.1017/S0022215120001450

**Published:** 2020-07-14

**Authors:** M Halliwell-Ewen, B Atkin, C T Huins, C L Dalton

**Affiliations:** Department of ENT, Queen Elizabeth Hospital Birmingham, UK

**Keywords:** Coronavirus, Otolaryngology, Otorhinolaryngologic Surgical Procedures, Outpatients, Pandemics

## Abstract

**Background:**

Coronavirus disease 2019 has demanded enormous adjustments to National Health Service provisions. Non-urgent out-patient work was initially postponed or performed virtually, but is now being re-established. In ENT surgery, aerosol-generating procedures pose a particular challenge in out-patient settings.

**Objective:**

A rapid restructuring of ENT out-patient services is required, to safely accommodate aerosol-generating procedures and increase in-person attendances, whilst coronavirus disease 2019 persists.

**Methods:**

Data were collected prospectively over four consecutive cycles. Two surveys were conducted. Results were analysed and disseminated, with recommendations for service restructuring implemented at cycle end-points.

**Results:**

Out-patient activity increased four-fold, associated with a significant rise in aerosol-generating procedures during the study period. Mean aerosol-generating procedure duration dropped weekly, implying a learning curve. Service restructuring occurred at cycle end-points.

**Conclusion:**

Iterative data gathering, results analysis and outcome dissemination enabled a swift, data-driven approach to the restructuring of ENT out-patient services. Patient and staff safety was ensured, whilst out-patient capacity was optimised.

## Introduction

Late January 2020 saw the first cases of coronavirus disease 2019 (Covid-19) in the UK,^1^ with the global outbreak being identified as a pandemic by early March 2020.^2^ The swift, exponential rise in cases and reported deaths throughout March 2020 ultimately required the UK government to adopt a nationwide disease suppression strategy.^3^ Sequential non-pharmaceutical interventions were rapidly introduced, to lower the reproduction number and significantly reduce viral transmission:^4^ self-isolation was followed by social distancing and school closures, public events were banned, and a country-wide lockdown was declared.^5^ Despite these measures, the UK has reported 289 140 confirmed cases and 40 883 deaths as of 10th June 2020.^6^

Covid-19 infection is caused by severe acute respiratory syndrome coronavirus 2 (SARS-CoV-2).^7^ It is highly contagious, spreading more readily than influenza.^8^ The virus spreads predominantly via small droplets originating from the upper respiratory tract during talking, coughing or sneezing.^8,9^ Aerosol-generating procedures are thought to pose a particular threat for healthcare workers.^10^ In part, infection severity is thought to be directly related to viral load exposure, with higher viral loads more likely to cause infection and more severe illness, as seen in influenza.^11^ The virus remains viable on plastic and stainless steel for up to 72 hours.^12^ Contaminated surfaces thus represent an additional, albeit subordinate, potential route of spread,^8^ but their consideration in healthcare settings is critical.

On 17th March, National Health Service (NHS) Trusts were instructed to postpone all non-urgent elective activity in order to maximise capacity to deal with the anticipated wave of Covid-19 cases, and to prevent the unnecessary exposure of non-infected individuals during routine elective activity.^13^ Consequently, advice was issued to convert non-urgent out-patient consultations into remotely conducted appointments where possible or, if not feasible, to postpone such activity.^14^ Face-to-face attendances were reserved for clinically urgent or emergency cases only. Management guidelines were published to minimise the exposure of healthcare workers and patients to Covid-19;^14,15^ these addressed personal protective equipment (PPE) and clarified new best practice.

Early reports following the Covid-19 outbreak in China highlighted ENT surgery and ophthalmology as amongst the highest risk specialties for medical staff to contract the infection.^16^ Established respiratory virus pandemic related guidance on infection control measures required to perform aerosol-generating procedures suggests that these procedures be conducted by the most experienced person, with minimum staff required, in a single room, whilst wearing full PPE.^17^ However, the evidence base for this remains controversial.^10^

In ENT, particular attention has focused on identifying which procedures should be classed as aerosol-generating procedures,^18^ thus requiring level 3 PPE.^9^ These include: flexible nasendoscopy, oral examination, aural microsuction, nasal packing and nasal cautery. On 29th April 2020, guidance was issued on how to re-establish elective services during the Covid-19 pandemic,^19,20^ whilst social distancing and stringent infection control requirements persist, and later updated.^21^

How to flexibly adapt services to ongoing changes in guidance whilst ensuring safe practice remains a continuing challenge during the Covid-19 pandemic. Here we report an iterative, four-cycle change in practice project, by which we restructured our ENT out-patient service to accommodate pandemic-related guidance in a data-driven manner.

## Materials and methods

We conducted a prospective quality improvement project examining the ENT out-patient service during the Covid-19 pandemic at the Queen Elizabeth Hospital Birmingham. It encompassed a literature review, four data collection cycles and two surveys.

A literature search of the PubMed and Cochrane databases was conducted (search 1 – ‘COVID-19’, ‘outpatient’ and ‘ENT’; search 2 – ‘COVID-19’ and ‘outpatient’; and search 3 – ‘COVID-19’, ‘outpatient’ and ‘aerosol generating procedure’). In addition, published guidelines from Public Health England, the Royal College of Surgeons of England and ENT UK were scrutinised for their relevance to out-patient clinic set-ups needing to incorporate aerosol-generating procedures during the Covid-19 pandemic.

The papers identified via the literature search were screened for relevance by title. Publications were excluded if they were unrelated to the organisation of out-patient services during the Covid-19 pandemic, or if they only contained non-specific infection control out-patient recommendations. The remaining articles were read in full to determine if they related specifically to the organisation of out-patient services during the Covid-19 pandemic and encompassed data on the timings of aerosol-generating procedures, or specifically addressed the challenges of maintaining patient flow in a department with a high volume of aerosol-generating procedures.

**Fig. 1.**
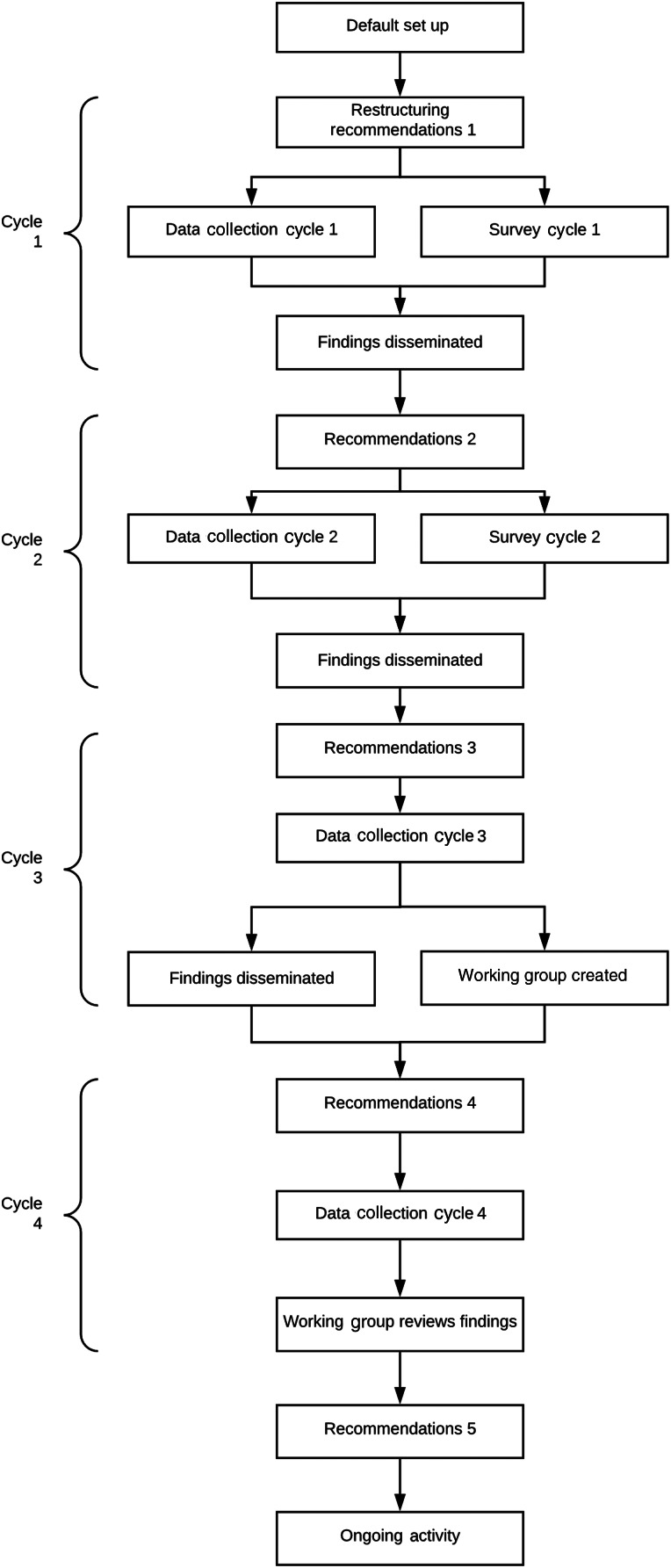
Flow diagram of iterative process used to restructure ENT out-patient set-up.

The four quality improvement project cycles each lasted one week (Figure 1). Initial analysis of the default ENT out-patient clinic set-up prompted consultation by the contributing authors with senior out-patient nursing, medical and infection control staff, which initiated a change in practice to accommodate infection control related issues surrounding aerosol-generating procedures (recommendations in cycle 1, Table 1). Data were collected prospectively from 21st to 27th April 2020 (cycle 1) to assess the adequacy of this new clinic set-up, and again from 1st to 7th May 2020 (cycle 2), 8th to 14th May 2020 (cycle 3), and 15th to 21st May 2020 (cycle 4). Electronic patient records were used to clarify missing data where required.

**Table 1.** Summary of cycle activity, including issues identified, recommendations made and outcomes achieved

Cycle	Issues identified	Recommendations	Outcome
1	Most ENT procedures are AGPs – Need separate room for AGPs – Need donning + doffing areas	*Allocate rooms:*	
1 × ‘cold AGP room’		Achieved but inadequate
1 × ‘hot AGP room’		Achieved but inadequate
1 × ‘donning room’		Achieved but not necessary
1 × ‘doffing room’		Achieved
Need to monitor new OPD set-up & activity	*Monitoring of OPD activity:*	
	Data 1 collection (clinic footfall & AGPs)	Achieved
	Survey 1 completion	Achieved
2	Not enough AGP rooms	*Increase number of procedure rooms:*	
		Allocate 5 procedure rooms	Achieved
		Only staff seeing in-person attendances to use clinic rooms	Partly achieved
		Emergency review clinics to be spread evenly throughout week	Not yet achieved, ongoing
		Adjust ratios of telephone:in-person slots	Not yet achieved, ongoing
		Even distribution of AGPs during day	Not yet achieved, ongoing
	Length of time required for AGPs	*Monitor timings for AGPS & post-AGP room clean:*	
		Midpoint co-ordinator to record timings on data collection sheet	Achieved
	PPE: lack of PAPRs	*Improve understanding of PAPRs issues:*	
		Assess junior doctors (*n* = 16) regarding PAPRs requirements	Achieved
		PAPRs to be collected daily from site office by junior doctor	Not achieved
	Ongoing need to monitor OPD set-up & activity	*Monitoring of OPD activity:*	
		Data 2 collection	Achieved
		Survey 2 completion	Achieved
3	AGP chaperone required	*Nursing staff training:*	
		FFP3 mask fit testing for all nursing staff	Partly achieved, ongoing
		PPE training for all nursing staff	Achieved
	PPE: lack of PAPRs	*Improve PAPR availability:*	
		PAPRs to be collected daily from site office by nursing staff	Achieved
		Procurement request for 2 dedicated ENT out-patient PAPRs	Requested, ongoing
	Lack of clinic & AGP rooms for re-starting elective out-patient clinics	*Clinic template adjustments:*	
		Even spread of emergency review clinic activity across whole week	Partly achieved
		Even spread of consultant clinic out-patient activity across week days	Not yet achieved, ongoing
		Consultant clinic template to include max. 3 × AGPs per session	Not yet achieved, ongoing
	Ongoing need to monitor OPD set-up & activity	*Monitoring of OPD activity:*	
		Data 3 collection	Achieved
	Restructuring of OPD services to include elective activity	*Working group:*	
		Set up with key members from ENT medical, nursing or infection control staff	Achieved
		Inaugural meeting within 7 days	Achieved
4	Clarification of post-AGP ‘room down time’ requirements	*Institute correct infection control measures for room preparation:*	
Assessment & advice from engineering & infection control		Achieved
Waiting room distancing needs may slow elective activity	*Aim for smooth ENT flow through out-patient department:*	
	Trust guidelines awaited regarding distancing constraints in ENT waiting area	Not yet achieved, ongoing
	Identify number of seats in waiting area allocated to ENT	Achieved, but may change
Clinic template adjustments ongoing	*Clinic template adjustments:*	
	Even spread of consultant clinic out-patient activity across week days	Partly achieved
	Consultant clinic template to include max. 3 × AGPs per session	Partly achieved
Ongoing need to monitor & restructure OPD activity	*Restructuring of OPD activity:*	
	Data 4 collection	Achieved
	Working group – weekly meetings until elective out-patient activity established	Ongoing
5	Clinic attendances rising & unclear ENT waiting room allowances	*Aim for smooth ENT flow through out-patient department:*	
		Trust guidelines awaited regarding distancing constraints in ENT waiting area	Awaited end cycle 5
		Identify number of seats in waiting area allocated to ENT	Awaited end cycle 5
		Working group chair to feed gathered ideas to clinical lead to pursue	Awaited end cycle 5
		Deconflict clustered clinic templates	Awaited end cycle 5
		*Clinic template adjustments:*	
		Even spread of consultant clinic out-patient activity across week days	Awaited end cycle 5
		Capacity calculations for 5 × AGP rooms by senior out-patient nurses	Awaited end cycle 5
		5 × AGP rooms to have individual clinic codes so AGPs can be booked directly	Awaited end cycle 5
	Emergency clinic procedure timings variable	*Emergency review clinic needs:*	
		Ring-fenced AGP room for emergency review clinic patients	Awaited end cycle 5
	Ongoing need to monitor & restructure OPD activity	*Restructuring of OPD activity:*	
		Data 5 collection	Awaited end cycle 5
		Working group – weekly meetings until elective out-patient activity established	Awaited end cycle 5

AGP **=** aerosol-generating procedure; OPD = out-patient department; PPE = personal protective equipment; PAPR = powered air-purifying respirator; FFP3 = filtering facepiece code 3

Data collected included the number of in-person out-patient attendances, and the number of aerosol-generating procedures performed. From cycle 2 onwards, data were collected on: the type of procedure performed (all types with a frequency of three or less were grouped as ‘miscellaneous’), aerosol-generating procedure duration, and whether the procedure was performed in the emergency review clinic (staffed by junior doctors) or a consultant-led clinic.

Room disinfection measures following the performance of an aerosol-generating procedure were conducted according to local hospital policy; specifically, a rest period (20 minutes), which allowed airborne particles to settle (determined by the number of ventilated air changes per hour per room), was followed by room disinfection (20 minutes).

Two e-mail surveys (surveys 1 and 2; Table 2) were sent to senior out-patient nursing staff and all members of the ENT medical staff, to obtain feedback on the restructured clinic set-up (36-hour response window). The data collection and survey results were disseminated to the surveyed staff via a socially distant governance meeting and e-mail (outcome dissemination 1 and 2), and later via a newly set up working group (outcome dissemination 3 and 4). New recommendations for further clinic restructuring (recommendations in cycles 2–5) were developed and implemented at the time of outcome dissemination, prior to further data collection.

**Table 2.** Questionnaires used in survey 1 and survey 2

Survey 1	Survey 2
Q1. What grade are you? (Consultant, middle grade, SHO/FY/PA, nursing)	Q1. What grade are you? (Consultant, middle grade, SHO/FY/PA, nursing)
Q2. Have you seen a patient in clinic, face to face, in the last 7 days? (Yes/no)	Q2. Have you seen a patient in clinic, face to face, in the last 7 days? (Yes/no)
Q3. If yes, were you aware that the clinic set-up had changed & how to undertake AGPs? (Yes/no/not applicable)	Q3. Was appropriate PPE, as per ENT UK guidelines, available to you either in clinic or from site office – if not, what was missing? (Comment)
Q4. Was appropriate PPE, as per ENT UK guidelines, available to you? (Yes/no/not applicable)	Q4. On which day(s) have there been issues with room availability to perform procedures? (Comment)
Q5. If you answered no to question 4 please list which items were missing: (Comment)	Q5. Compared to the previous week, has there been any change to room availability? (Comment)
Q6. On which day(s) have there been issues with room availability to perform procedures? (Comment)	Q6. Have you been able to move virtual clinics to another location? (Yes/no/not applicable)
Q7. What comments do you have on the overall system, especially regarding problems/causes? (Comment)	Q7. In the last week, how often have you required nursing staff to be present in the procedure room whilst performing AGPs? (None of the time/ occasionally/ most of the time/ all of the time)
Q8. What improvements/modifications to the clinic system would you suggest as we look to start re-introducing some more usual clinic activity whilst still needing to accommodate potentially positive Covid-19 patients? (Comment)	Q8. Looking ahead, do you anticipate needing nursing staff to be present in the procedure room whilst performing AGPs? (None of the time/ occasionally/ most of the time/ all of the time)
	Q9. What comments do you have on the overall system, especially compared to last week? (Comment)

SHO = senior house officer; FY = foundation year doctor; PA = physician assistant; PPE = personal protective equipment; AGP = aerosol-generating procedure; Covid-19 = coronavirus disease 2019

Spearman's rank correlation was used to evaluate correlation between in-person attendances and the number of procedures performed. Paired *t*-tests were used to evaluate differences between mean timings according to the type of procedure performed and the type of clinic that the procedure was performed in. Statistical analyses were carried out using MatLab2019a.

## Results

The literature search identified 316 articles, of which 299 were excluded after screening by title. Seventeen articles were identified as potentially relevant and scrutinised in full. Of these, 15 were excluded as they did not cover out-patient clinic set-ups encompassing aerosol-generating procedures during the Covid-19 pandemic. The remaining two publications described ENT clinic models incorporating aerosol-generating procedures.^22,23^ However, neither publication considered out-patient models based on data that included aerosol-generating procedure duration, nor specifically addressed the challenges of maintaining patient flow in a department carrying out a high volume of aerosol-generating procedures.

The iteration cycles performed during our study period included: four data collection episodes, two surveys, four points of outcome dissemination and five sets of recommendations (Figure 1). Throughout the study, in-person attendances increased four-fold between cycle 1 and cycle 4, whilst the amount of aerosol-generating procedures performed rose by 463 per cent (Table 3). Out-patient attendance was greatest on Thursday in cycle 1 (40 per cent of attendances), Wednesday in cycle 2 (29.9 per cent), Wednesday in cycle 3 (31.1 per cent) and Thursday in cycle 4 (32 per cent). Aerosol-generating procedures were most frequently performed on Thursday in cycle 1 (62.5 per cent), Friday in cycle 2 (24 per cent), Monday in cycle 3 (28.6 per cent) and Thursday in cycle 4 (27 per cent). Overall, there was a statistically significant correlation between in-person attendance and the number of procedures performed (*p* < 0.001).

**Table 3.** In-person out-patient attendances and aerosol-generating procedures performed per cycle

Week day	In-person attendances (*n*)				AGPs performed (*n*)			
	Cycle 1	Cycle 2	Cycle 3	Cycle 4	Cycle 1	Cycle 2	Cycle 3	Cycle 4
Monday	1	8	13	18	0	5	6	9
Tuesday	1	8	20	5	0	1	3	4
Wednesday	4	20	28	32	0	3	3	7
Thursday	10	16	20	33	5	4	4	10
Friday	8	15	5	11	2	6	2	2
Saturday	0	0	1	7	0	0	1	5
Sunday	1	1	3	1	1	1	2	0
Totals	25	67	90	100	8	25	21	37

AGP = aerosol-generating procedures

Survey 1 had a 64.3 per cent response rate (18 out of 30, comprising 7 consultants, 4 middle-grade doctors, 6 senior house officers or foundation year doctors, and 1 senior nurse). Of the respondents, 77.8 per cent (14 of 18) had seen patients in person during cycle 1, and 93 per cent (13 of 14) had access to PPE as recommended. One respondent could not access the powered air-purifying respirator, requiring a colleague (successfully fit-tested for a filtering facepiece code 3 (FFP3) mask) to perform that aerosol-generating procedure. Seven of the 14 respondents had problems finding a room to perform an aerosol-generating procedure, all of whom identified day 3 (Thursday) in cycle 1 as the problem day. Responses to question 7 in survey 1 identified a lack of room availability for aerosol-generating procedures, with concerns that this may become a greater problem once elective activity resumes (eight respondents). Answers to question 8 in survey 1 suggested that more aerosol-generating procedure rooms were needed (four respondents), with separate Covid-19 negative and positive areas being maintained (three respondents). Complete responses can be viewed in Appendix 1.

Findings from the first data collection episode and the first survey were disseminated and discussed at our clinical governance meeting (outcome dissemination 1), from whence recommendations for further clinic restructuring (recommendations in cycle 2) were compiled (Table 1). Of note, the first data collection episode and the first survey identified that cycle 1, day 3 (Thursday) had been the most problematic, with the most attendances, the most aerosol-generating procedures and a reported acute shortage of available rooms to perform aerosol-generating procedures. Analysis of the attendance times identified that all patients requiring aerosol-generating procedures (*n* = 5) had attended in the same 90-minute window, on a day when several parallel head and neck cancer clinics took place.

Following consultation with departmental staff, five rooms were made available for aerosol-generating procedures (instead of two), and ring-fenced as ‘procedure rooms’. The hot and cold room set-up for aerosol-generating procedures was abandoned, as local infection control advice identified that all rooms used for aerosol-generating procedures require identical cleaning procedures irrespective of a patient's Covid-19 status. The donning room was also dropped, as PPE can be donned in the procedure room prior to performing an aerosol-generating procedure.

It was identified that aerosol-generating procedure data were needed to enable appropriate forward planning. This was included in the recommendations for cycle 2. Subsequent recommendations are detailed in Table 1, including outcome status at the time of publication. Overall, 33 change in practice recommendations were made during cycles 1–4, of which 60.1 per cent (20 of 33) have been achieved, 15.2 per cent (5 of 33) have been partly achieved and 24.2 per cent (8 of 33) remain to be achieved at the time of writing.

Data on aerosol-generating procedure durations were collected for 52 per cent, 90.5 per cent and 97.3 per cent of aerosol-generating procedures in cycle 2, cycle 3 and cycle 4, respectively (Table 4). The reduction in mean aerosol-generating procedure duration between cycle 2 and cycle 4 was statistically significant (*p* = 0.0250); however, no significant difference existed between cycles 2 and 3 (*p* = 0.101), or between cycles 3 and 4 (*p* = 0.903). The range of aerosol-generating procedure duration remained similar for the different cycles. Table 5 shows the overall number of aerosol-generating procedures according to clinic type, for which timings had been recorded during cycles 2, 3 and 4. Overall, data on aerosol-generating procedure duration were collected for 37 of 54 (69 per cent) and 31 of 41 (76 per cent) of aerosol-generating procedures performed in the emergency clinic and consultant-led clinic, respectively. There was no statistically significant difference between the mean aerosol-generating procedure duration in the emergency clinic versus consultant-led clinic (*p* = 0.204). Table 6 depicts the types of aerosol-generating procedures performed according to procedure type, with no statistically significant difference in mean aerosol-generating procedure duration.

**Table 4.** Aerosol-generating procedures performed in cycles 2–4, with duration details

		AGP duration (minutes)		
Cycle	Number of AGPs	Mean	Median	Range
2	13	37	30	10–90
3	19	25	17.5	7–70
4	36	16.2	12	2–80
Totals	68	26	18.5	2–90

There was a statistically significant difference in mean aerosol-generating procedure duration between cycles 2 and 4 (*p* = 0.0250). AGP = aerosol-generating procedure

**Table 5.** Aerosol-generating procedures performed during cycles 2–4, with duration details, according to clinic type

		AGP duration (minutes)		
Clinic	Number of AGPs	Mean	Median	Range
Emergency review	37	26	22.5	2–90
Consultant-led	31	20	14	4–70
Totals	68	26	18.5	2–90

There was no statistically significant difference in mean aerosol-generating procedure duration between clinic types. AGP = aerosol-generating procedure

**Table 6.** Aerosol-generating procedures performed during cycles 2–4, with duration details, according to procedure type

		AGP duration (minutes)		
Procedure performed	Number of AGPs	Mean	Median	Range
Flexible nasendoscopy	29	21	15.5	2–90
Microsuction	22	23	20	5–90
Nasal packing & cautery	4	31	17.5	8–80
Miscellaneous	10	32	29	4–68
Missing data	3	–	–	–
Totals	65	24	17	2–90

There was no statistically significant difference in mean aerosol-generating procedure duration between the procedure types performed. AGP = aerosol-generating procedure

Survey 2 had a 53.3 per cent response rate (16 out of 30, comprising 3 consultants, 7 middle-grade doctors, 5 senior house officers or foundation year doctors, and 1 senior nurse). Of these, 14 had seen patients during the data collection period. One of 14 respondents had difficulty finding a room in which to perform an aerosol-generating procedure. Free-text answers are outlined in Appendix 2.

Following cycle 2, we identified that 47.4 per cent of junior doctors (9 of 19) had failed fit-testing for FFP3 masks, thereby requiring a powered air-purifying respirator in order to have adequate PPE. Thus, subsequent recommendations included that powered air-purifying respirators should be readily available in the ENT out-patient department (Table 1).

During our study period, the number of ENT ring-fenced seats in the out-patient waiting room was reduced from 15 (pre-pandemic) to 4 (26.7 per cent). At the end of cycle 4, this was raised as a potential rate-limiting step, as footfall had started to increase, influencing subsequent clinic template proposals.

## Discussion

Managing an out-patient department during the current Covid-19 pandemic poses new challenges, particularly in high-risk specialties such as otolaryngology,^24^ where aerosol-generating procedures are performed frequently. Covid-19 related guidance stresses PPE requirements for aerosol-generating procedures,^18,24,25^ including how PPE should be donned and doffed.^26,27^ However, most NHS ENT out-patient departments are not set-up to easily accommodate infection control measures related to respiratory pandemics. Early recognition that safe performance of aerosol-generating procedures would pose a particular challenge for our out-patient services during this pandemic initiated the iterative change in practice approach reported here. This enabled rapid, data-driven restructuring of our out-patient set-up, ensuring the safe performance of otherwise routine ENT procedures.

To date, minimal literature reporting on out-patient services in the Covid-19 pandemic exists. Lal *et al*.^28^ produced recommendations for an orthopaedic out-patient department. They adjusted their clinic set-up with respect to social distancing requirements, and published a floor-plan and patient flow model. They estimated the likely maximum clinic capacity from clinicians’ estimations of procedure duration,^29^ but did not report collected data to support and refine these changes. Additionally, the orthopaedic clinic did not routinely perform large numbers of aerosol-generating procedures and, thus, planning for these did not form part of their work.

Our literature review identified that, to date, little specific evidence exists regarding: the performance of aerosol-generating procedures, out-patient footfall, and the incorporation of infection control measures relating to Covid-19 within an ENT out-patient setting. Of the two articles identified as relevant, Weiss *et al*.^23^ describes changes to the set-up of their out-patient ENT department, including the double triage of patients into ‘hot’ and ‘cold’ streams, to minimise room contamination. They integrated this into an ‘operational concept’ and implemented it within 48 hours following approval from infection control teams. They alluded to their ‘operational concept’ having been produced by an iterative process, but did not publish the structure of this process, or evidence of cyclical data collection, recommendations and feedback. In contrast, Lescanne *et al*.^22^ produced a stepwise guide to the re-organisation of ENT out-patient services, which included changes to their triage area, waiting area and reception area, to minimise contact between patient and staff. They identified that common ENT procedures would produce infectious aerosols, requiring stringent PPE and disinfection, but did not examine the unique space and time constraints posed by high volumes of aerosol-generating procedures in an out-patient department. Neither of these published studies reported an iterative approach to out-patient clinic structure that was driven by recorded attendance and timing data.

Overall, the iterative approach we used enabled the formulation of 33 recommendations during the study period. The majority of these had been achieved by the time of writing (60.6 per cent), most within the cycle subsequent to the identification of the issue.

Our results demonstrate a four-fold increase in face-to-face attendances during the period studied. This may reflect patients’ initial relative fear of attending hospitals towards the beginning of lockdown, which has been subsiding as reports of new Covid-19 infections and deaths abate. As expected, we found a statistically significant correlation between the number of in-person attendances and aerosol-generating procedures performed. Whilst recent months have seen an increased use of telemedicine and virtual clinics to accommodate elective out-patient activity, this may decrease as elective face-to-face services are resumed.^19–21^ Particularly in ENT, where remote patient examinations are challenging, out-patient departments need to expect an ever-increasing footfall and prepare to accommodate the growing number of associated aerosol-generating procedures.

Interestingly, our study highlights that different factors can limit the number of clinic attendances an ENT department will be able to accommodate. One rate-limiting step identified was the significantly reduced number of seats in our waiting area because of distancing requirements (4 instead of 15 seats). Another was the finite number of procedure rooms available for performing aerosol-generating procedures (*n* = 5).

Our results also identified a mean aerosol-generating procedure duration of 26 minutes overall, which dropped from a mean of 37 minutes (in cycle 2) to a mean of 16 minutes (in cycle 4). This statistically significant reduction is highly unlikely to be related to a sudden increase in clinicians’ speed at performing the procedures. Instead, we suggest it indicates a learning curve effect regarding donning and doffing PPE, as well as a more efficient use of procedure rooms for aerosol-generating procedures. Contrastingly, no significant difference in mean aerosol-generating procedure duration was identified between nasoendoscopy, nasal packing and aural microsuction, indicating that the turn-around time for procedure rooms is similar irrespective of the nature of the aerosol-generating procedure performed. Additionally, we found no statistically significant difference in aerosol-generating procedure duration in the emergency clinic versus consultant-led clinic.

Overall, our data have enabled a single timing figure to be used to plan all of the proposed clinic templates. Following aerosol-generating procedures, a procedure room must ‘rest’ before it is safe to clean.^9^ This rest period is dependent on the number of air circulations.^9^ In our department, a procedure room requires a 20-minute rest period, followed by a 20-minute disinfection period. Consequently, our procedure rooms are ‘occupied’ for a mean of 56 minutes (equating to the mean aerosol-generating procedure length plus the rest period plus the disinfection period) for every aerosol-generating procedure performed. This total ‘room time’ directly determines the number of aerosol-generating procedures that can be performed in one procedure room per session, which will be significantly reduced compared to pre-pandemic sessions. Additional factors may impose further restrictions to patient throughput and will need to be assessed individually for each department.

- Aerosol-generating procedures represent a unique challenge to ENT out-patient services during the coronavirus disease 2019 (Covid-19) pandemic
- This is the first report describing data-driven changes to ENT out-patient services during a respiratory pandemic
- Analysis of rate-limiting steps enabled development of a safe, workable, Covid-19 out-patient model
- A multidisciplinary approach is paramount to ensure successful, rapid introduction of service changes
- Iterative approaches to practice changes allow pandemic-related issues to be addressed swiftly and flexibly

Most clinicians are familiar with the clinical audit cycle. A clinical audit's critical feature is a comparison to published best practice.^29^ Whilst there are useful published guiding principles for social distancing and cleaning in an out-patient setting,^9,18,22,28,30^ there is no replicable ‘gold-standard’ model relating to aerosol-generating procedures within the out-patient setting during the Covid-19 pandemic. Thus, our work does not fit the traditional definition of a clinical audit cycle. Instead, early recognition that our clinic set-up would need to accommodate aerosol-generating procedures led to initial clinic restructuring recommendations, which catalysed an ongoing, flexible and radical change in practice, underpinned by an iterative approach. The Association of Qualitative Research describes the iterative approach as ‘particularly useful for time-sensitive projects where there is not scope for multiple rounds of research’.^31^ We posited that restructuring our set-up early, and responding to issues as they arose, would enable us to remain abreast of ongoing changes to practice guidelines.

Our approach shares some key concepts found in the ‘agile’ methodology common in current management doctrine,^32^ which stresses a short-cycle process of implementing changes, followed by testing and rapid feedback to correct issues. Tolf *et al*.^33^ examined the application of agile concepts to hospital management, reporting that agile approaches are underpinned by a capacity to flexibly respond to both organisational and environmental variability. The high degree of unpredictability frequently seen in hospital settings allows the agile approach to be of great value for changing hospital management processes. We suggest that in volatile circumstances, such as the current pandemic, an agile approach also provides a highly constructive strategy with which to address clinical processes in the face of rapid change.

## Conclusion

The Covid-19 pandemic has brought unprecedented changes to healthcare systems globally. In otolaryngology, aerosol-generating procedures comprise a common, necessary part of out-patient activity, posing a particular challenge during this pandemic. As elective out-patient work resumes, every ENT department needs to consider how to accommodate increasing numbers of aerosol-generating procedures with minimum risk of infection for patients and staff. Currently, no literature exists to directly address this challenge.

Our four-loop quality improvement project incorporated principles found in iterative approaches common to agile models used in business. This allowed real-time, creative adaptation of ENT out-patient services to introduce pandemic-related changes. It enabled early recognition that aerosol-generating procedures now take far longer to complete, which impacts out-patient activity. It also allowed us to accommodate a four-fold increase in footfall and related aerosol-generating procedures in our service. We recommend this methodology for situations where unexpected, rapid out-patient service adjustments are required. We suggest data collection starts simply, following the first out-patient restructuring activity. It should cover aspects such as out-patient footfall trends, room and waiting area availability, and timings of possible rate-limiting steps. The cyclically gathered data should then be assessed in a multidisciplinary setting consisting of relevant stake-holders. This facilitates a unified approach for subsequent practice adjustments. The whole process should continue until an optimum steady state of practice has been reached.

Our findings demonstrate a constructive, rapid and successful process with which to tackle pandemic-related service challenges, without breaching the guiding principles that protect patients and staff.

## Appendix 1. Complete set of responses to survey 1, questions 7 and 8

**Table:**

What comments do you have on the overall system especially regarding problems/causes?	What improvements/modifications to the clinic system would you suggest as we look to start re-introducing some more usual clinic activity whilst still needing to accommodate potentially positive Covid-19 patients?
‘Not enough AGP rooms’	‘More AGP rooms, nursing staff outside “hot” rooms’
‘I think the system is responsive to our changing needs – so I have no problems’	‘Having a Covid-negative facility to see patients’
‘Large number of cons clinics on same morning – 3 AGPs performed all at same time meaning a 4th AGP from emergency clinic could not be performed. Patient had to wait 2 hrs’	‘Spread cons clinics across days to avoid rooms not being available’
‘If the routine/elective clinics are to be set up, I envisage very limited number of patients who can be seen in the present layout. As we need to FNE in few patients, the clinicians would be queuing to see their patients. This layout will work with one or two maximum clinics at a given time’	‘To enable more patients seen, number of rooms required would be more. But given the logistics, the one option would be to run three sessions so that the patients can be seen keeping the same layout but spacing the sessions and may be even considering 7 day service!’
‘I haven't seen any patients!’	‘Please give an induction’
‘There was no induction to the new format’	‘More examination rooms’
‘Effective system’	‘Main issues are ensuring the timely availability of clinic rooms and that rooms are properly sterilised in between patients. Currently I feel that there is enough capacity during the day to accommodate more clinic patients (as the SHO clinic list now runs into the afternoon)’
‘Need to make audiology aware in advance, otherwise works well. But in the emergency clinic, SHO is a bit redundant as it is now reg led!’	‘Reduced clinics and patient numbers as many will need examination and treatment requiring the use of PPE’
‘Just examination room availability’	‘Maintain Covid hot area and cold areas’
‘The introduction of donning and doffing rooms, in addition to “hot” and “cold” clinic rooms means that if there are other clinics (e.g. H&N) running in conjunction with the SHO clinic, there may be delays/an inadequate number of rooms to review patients in a timely manner’	‘Space in waiting rooms, hot and cold areas’
‘Working well for head and neck. Will be problems with having to wait 20 mins for rooms to be usable after AGPs’	‘Maybe leave bigger gaps between patients in SHO/all clinics to accommodate the down time after AGPs. That would keep other rooms free’
‘Room availability due to rooms having to be closed post AGPs’	Need to be mindful of the cleaning down of rooms, in between patients; the room will be out of use for a minimum of 30 minutes
‘We need to spread out the times and days of seeing patients, Thursday in particular is busy in terms of room use. We have extended the review pt clinics to include Saturdays and Sunday; this is not being utilised at the moment. We can set up the review clinic to run in the afternoon also. This will spread the workload throughout the day and over 7 days’	‘Initial telephone consultations to try to ascertain those patients who are unlikely to need an AGP, so that they can be seen either in another clinic room or built into the clinic model alongside AGPs (see another patient whilst waiting for the AGP room to become clean again)’
‘Flow through the department will be significantly reduced due to the slow turn around for AGP rooms’	‘As above, be aware of rooms for procedures and how long after they are unavailable’
‘Spread out the patients who will need procedures. We are working a 7 day service’	

Covid-19 = coronavirus disease 2019; AGP = aerosol-generating procedure; cons = consultant; hrs = hours; FNE = flexible nasendoscopy; SHO = senior house officer; reg = registrar; H&N = head and neck; mins = minutes; pt = patient

## Appendix 2. Complete set of responses to survey 2, question 9

**Table:**

What comments do you have on the overall system, especially compared to last week?
‘Slow to see patients and very limited clinic space’
‘I think that there should be more than one person in the room when doing AGP’
‘Much improved’
‘People are more aware of the regulations’
‘Good to have more procedure rooms available’
‘Seems slicker overall’
‘N/A – have not used clinic since new system implemented’
‘Slicker; doctors know system better and appreciate need for warning. Need to doff gown in room, and close room’
‘Better’
‘Very good organisation of the ENT out-patients’
‘Haven't been in clinic this week’
‘Unable to comment as I am on nights this week and therefore unable to compare’
‘Time saving’
‘Competent’
‘New issue raised of waiting room capacity and flow’
‘First week of clinic’

AGP = aerosol-generating procedure; N/A = not applicable
